# On Malingering

**Published:** 1908-10-17

**Authors:** Seymour Taylor


### Hospital Clinics.

### ON MALINGERING.

By SEYMOUR TAYLOR, M.D., F.R.C.P.

(A Lecture given at the West London Post-Graduate College.)

I have chosen this subject for to-day's lecture owing to the fact that some of you may have to support by skilled evidence a claim for injury, or to expose the falsity of a supposed injury. Others being engaged in his Majesty's services must at times come across cases in which malingering is difficult to detect, and yet imperative to demonstrate. It will also be necessary that I should include some consideration of hypochondriasis, of neurasthenia, and of hysteria.

True malingerers are found for the most part in the lower ranks of society, in the Army and Navy, and amongst prisoners, though I must qualify this last statement by saying that some of the most clever malingerers, men who meet an astute question with an equally astute and evasive answer, are to be found in criminals who have brought themselves within the four walls of a prison by reason of their over-active brain-energy being directed into crooked courses.

In recent years, and up to a certain date, there can be no doubt that malingering was less frequent than it used to be. This may be accounted for by the less frequent and mitigated sentences of corporal punishment which were at one period regarded as necessary for the true maintenance of discipline; by the shortened lengths of service; and possibly by the knowledge on the part of the would-be malingerer that scientific methods of detection are now used by the medical officer. On the other hand, in civil practice, it is quite probable—and, so far as my experience goes, it is certain—that, owing to the effect of recent legislation, malingering is on the increase. Again, experience tells us to be on our guard when any one of the better middle and upper class is the subject of inquiry as to injury. Only let a railway company, or a rich corporation, be the object of attack, and it will be surprising how an apparently trivial injury, say, to a professional man, will cause prolonged incapacity and distressing symptoms, all of which disappear shortly after the trial is over and the award shall have been made, no matter whether that award was more than or less than was expected or contended for. It will thus be seen that, so far as my experience goes, malingering, though most frequently met with in adult males of the lower classes, is not really limited to any rank of society, or to any age, or to either sex.

Subjective symptoms are difficult to diagnose, and therefore difficult to controvert. A man, after some slight accident, says that he has constant or frequent headaches, and it is impossible for a medical man to deny his statement; but short of injury or mutilation, the impostor's ingenuity, especially in that class which is composed of ignorant people, is distinctly limited. But there is one rule which I lay down, for myself at any rate, and it is this: Although one's reputation as a skilful medical man may be at stake, it were better to be taken in over and over again than to make a grievous error detrimental or injurious to the complaint.

Let me first say a few words to the Civil practitioners in my audience. I have now had considerable experience in examining claimants for compensation for injuries received in railway and other vehicular accidents, and also workmen who have received injuries, some severe, others mostly trivial. And as they all claim damages, especially under the provisions of recent Acts of Parliament, it is highly necessary that you should on the one hand insist on proper remuneration in undoubted permanent injury, whilst on the other you should be enabled to expose fraud and convince a tribunal. I may say at the onset that I know of no legislation which has tended to promote fraud so much as the Acts of Parliament which I have in mind. In the great majority of cases, though by no means in all, two circumstances have impressed themselves on my observation—namely, (1) the trivial character of most of the accidents and the small amount of injury sustained; (2) the ages of the injured claimants.

1. It is not a little surprising how often a small accident, such as a slight fall, a rick in the back, or a cut scalp, is followed by the subsequent narration of symptoms which can in no way be accounted for by the injury, and which are purely subjective and not confirmed by the co-existence of any signs or other evidence which medical science can detect. Indeed, certain symptoms are often said to exist which cannot be explained on either anatomical or physiological grounds, and only therefore rest on the statement of the complainant. Counsel seize hold of these statements, and it requires some considerable knowledge and skill on the part of the medical witness to controvert them and to expose their fallacies. The claimants in some instances are distinctly malingering; in others they imagine their pains and disabilities, but become speedily cured after their cases have been settled, one way or another.

2. In my experience the great majority of those who claim weekly compensation under the recent Workmen's Compensation Acts are men past the meridian of their lives, men whose powers of activity and whose wage-earning capacities are on the wane. Obviously the powers of recovery in such men are not so great as in younger people. Still, I am convinced that there are now more applicants for compensation for lengthy disablements than there used to be, and it would be interesting to ascertain whether statistical figures support this view.

Though wishing to keep an unbiassed mind on the subject, I cannot help thinking that many complaints of injuries and subsequent disabilities would never be mentioned or brought to light were it not for the knowledge that compensation is easily claimed and in many instances equally easily obtained.

I find two distinct and separate classes of claims.

One class, mostly young men, claims a large sum in compensation rather than a weekly dole. The large sum frequently allows them to start in some business or enterprise, and relieves them of the monotony of manual labour. A weekly sum of some eighteen or twenty shillings is not enough for a man of this class to support himself and his family on. A sum of one hundred pounds or more makes him a capitalist, and excites his desire for fresh avenues of livelihood. On the other hand, a man of sixty or thereabouts has passed the zenith of his activity and his wage-earning capacity. He finds himself being pushed aside by younger and more active men. His family is probably grown up and self-supporting, and his own wants now being fewer, he can live in comparative ease on, say, seventeen shillings a week, an award which is wealth when compared with the miserable pittance which the State can afford.

But do not for a moment think that I am adverse to an injured man receiving due compensation. An injury must be recompensed and paid for; and many employers bring trouble on themselves by resisting honest claims. Further, a severe or permanent injury is not balanced by monetary payment, however large that payment may be. My object in this lecture is to endeavour to show you how to detect the fraudulent claimant, but at the same time to assist the honest one.

There is yet, as I have said, a class of people who, though often suspected of malingering, are malingerers only in part. They belong to the neurotic class; they are the representatives of hysteria in women, and not infrequently are of the educated and executive ranks. To begin with, they are for the most part hypochondriacs, or valetudinarians, a people of unstable nervous system. Any pressure in work, any financial worry, any family trouble, or disappointment renders them temporarily unable to follow their occupation. They have all sorts of fears and dreads, mostly without foundation, and demand long periods of rest, on full pay.

In the early stage they are mimics or exaggerators, having some slight ailment or distress. And if taken in hand at this early stage, they may be run out of their grooves into healthy channels, and their fancied ailments and pretended distresses laughed out of court, they themselves joining subsequently in the levity. But beware here of neglect. A fancy may become a fixed idea, and without tact and judicious treatment on the part of the medical adviser, the magnified trivial ailment may become a confirmed conviction. A partial malingerer may show signs of mental aberration, and the end of the chapter may be insanity.

It is somewhat difficult to classify malingerers; but the following is perhaps as good a classification as can be made:—

(*a*) Some, though complaining much, have no signs or symptoms of disease or injury. They are liars pure and simple. They constitute the class known as fictitious malingerers.

(*b*) Others, again, purposely produce sores or injuries and magnify their sufferings; they are rogues, and come under the category of factitious malingerers.

(*c*) Others, again, though suffering from slight injury, are such mental perverts that they make the most of their troubles, which are to them a stock-in-trade. They are exaggerators and romancers.

(*d*) There is yet a fourth class in which malingering and neuro-mimesis go together. They are not entirely malingerers, but are people of unstable nervous system. The advent of some injury or trouble upsets their mental equilibrium, and convulsions, fits, and other neurotic manifestations are produced almost at command, and always with very little excitement or provocation.

In almost all cases of malingering there is some motive, such as the evasion of punishment in prisons and irksome duties in the Services, the premature acquisition of pensions or retiring allowances, the greed of gain ensuing from accidents or illnesses which are covered by an insurance policy, nourishment and support for the idle by the charitable public, and a yearning for affection or sympathy. It may, however, in some cases be impossible to assign any motive. The patient is often on the border land of insanity, and should be so treated.

It would be impossible in the time which is devoted to one lecture to speak of the many forms of malingering which may be met in the Royal Services. And probably some of you are greater authorities than I on this subject, having had more experience. But in my endeavours to put you on your guard against possible imposture, I will mention some few lesions which are most frequently simulated; and it will perhaps be best to arrange them on an anatomical basis.

Ulcers are easily produced by mechanical means, and sometimes by chemical agency. As a rule an ulcer due to disease is a chronic lesion, with indurated irregular edges and an indolent tendency to granulation. In similar lesions caused by chemicals, detection may often be made by examining the discharge on the covering bandages for some preparation of copper or of potassium or of silver nitrate, or the like.

In every case of supposed injury to bone or joint insist on removing all bandages, and carefully examine the injury, engaging the attention of the patient whilst so doing by some trivial question or conversation. A pretended fixed joint may then be "accidentally" moved or forcibly flexed without causing pain or demur. In pretended fractures or dislocations an examination by the aid of *x*-rays will give conclusive evidence.

Impostures in connection with the circulation are more difficult to expose than one would imagine. Palpitation may be produced by taking cordite, the explosive used in our military cartridges. It is also brought on by an astute malingerer taking some violent exercise immediately before his visit to the doctor. But œdema is perhaps the most common subterfuge. A careful examination of the heart and lungs with negative results should make you suspicious, especially if the man should have no attendant dyspnœa. In true œdema dyspnœa is, in the vast majority of cases, an antecedent symptom.

A feigned cough is overdone; a true cough is frequently increased by speaking or reading aloud. Hæmorrhage may proceed from the gums, and is then mixed with saliva. If from the lungs it is frothy and contains epithelium from the respiratory tract; then a careful auscultation of the lungs should reveal its source of origin. In all instances the ratio of pulse-rate to respiration should be carefully noted.

The gastro-intestinal tract is a fertile field for imposture. Diarrhœa may be asserted, or actually produced by the self-administration of drastic purges. But in some instances a constipated evacuation has been broken up in some urine. It is always well, therefore, to counter this ruse by compelling the malingerer to use separate utensils for urine and for fæces, and, if possible, secure an attendant to be present when evacuation takes place, and inquire from him as to the passage of flatus, which nearly always occurs in true diarrhœa. In assumed involuntary defæcation the examination of the lower (voluntary) sphincter may detect the presence or absence of spasm; the latter condition almost invariably attends incontinence.

Vomiting may be induced artificially by well-known methods. Detection of fraud may generally be made by noting the time of its occurrence, its association with, or independence of, food. In case of difficulty the patient can easily be watched unknown to him.

In all cases of assumed rupture, unless of recent date, the wearing of a truss leaves a depression over the outlet with a staining of the skin. Obviously every aperture through which the bowel can emerge should be carefully examined.

Fraudulent jaundice is perhaps the most easy of all impostures to detect. If of the non-obstructive type, there will surely be the history of some antecedent fever or grave constitutional disease. If of the obstructive type, one would find a clayey stool, a highly stained urine, and yellow conjunctivæ. All these three clinical signs can seldom or never be counterfeited together.

Genito-urinary diseases are a frequent pretext for avoidance of duty or punishments. If incontinence of urine be assumed, one's suspicions should be aroused if the glans penis and prepuce are not sodden and swollen and if the bedding is free from pollution. In pretended albuminuria or glycosuria the shortest way to detect fraud is to insist on the examination of a catheter specimen of urine, since the opportunities of submitting substituted urines, or a urine to which white-of-egg, blister fluid, blood, sweetmeats, or other contaminations have been added, are easily found by the impostor.

Varicocele occupies a debated or controversial field. Many candidates for the Services, whether civil or military, who have varicocele are rejected by the medical examiners, whilst the existence of a varicocele is often eagerly welcomed by the malingerer. As regards the first proposition, no general rules can be laid down applying equally to all cases and to all ranks. But although somewhat outside the scope of this lecture, I may be permitted to tell you that in some instances men may be admitted with varicocele to the Services without any risk, whilst in others the existence of a varicocele should always be regarded as a suspicious predisposing cause of malingering. For it must be admitted that whilst we cannot deny the existence of pain when its presence is asserted, we must also admit that varicocele is frequently attended by an incapacitating anguish.

Roughly, I would say that varicocele in an officer may be overlooked, provided it is not a severe one and that he is a good horseman. Varicocele if at all severe calls for surgical treatment in a candidate for a civil appointment in which horsemanship is required—my experience telling me that civilians who seek Government appointments are poor equestrians, and are therefore more liable to receive injury to a varicocele.

In the lower branches of the Services our attitude should be different. I advise rejection of a candidate for the Army or Navy if he should be found to suffer from varicocele to any appreciable degree. It may not only actually incapacitate him for strenuous duty, but it may act as a convenient peg on which to hang complaints and excuses for shirking duties, and even demands to escape foreign service. In such men a mild degree of varicocele is more serious than a more extensive one in educated and better bred men.

Assumed deafness may sometimes be exposed if the sound ear be covered up and a conversation begun in a loud voice on some interesting topic. As the malingerer becomes more intent on the conversation, your voice may be gradually lowered, and then you may ask a leading question in a subdued tone, often receiving a reply which is conclusive that the malingerer has heard you. A similar trial may be made even when deafness is supposed to affect both ears.

Blindness, if pretended, had best be detected by secretly watching the man. There is no language or expression in the eyes of a blind man, and it will probably soon be discovered that instead of having the stony reptilian stare, he is looking at different objects, though they be close together. A careful ophthalmoscopic examination will considerably help to elucidate any fraud. I do not advise you, as was formerly done in cases of pretended blindness, to march your man to a precipice or to a river's edge. Think of the dismay if the trial did not end successfully for the medical examiner.

In the investigation of palsies one or two points are useful to bear in mind. Make your visit for examination at an unexpected time. Always have the condemned limb and its fellow equally exposed, all wraps and bandages being removed. Assumed tremors and rigidities and deep reflexes will be grossly exaggerated and overdone, the latter especially so. They are not infrequently produced before the eliciting tap is made, the blow being almost reflexly anticipated by an "old hand." It is often advantageous to have a mirror in the room, which should be conveniently placed for reflected observations. Occasionally, and especially with veterans, a short, sharp word of command will restore a palsied limb or cure a deafness.

In all examinations when deceit is suspected be bold, but not brusque, in your methods of examination. Make no hurried diagnosis, and never proclaim your opinion to any but the proper authorities.

It is better at first to pretend to believe. Deceit is often best encountered by subterfuge; meet feigning by feigning. Always bear in mind that signs are for the most part overdone. The *facies* of a malingerer is one of sorrow mixed with pain, the former predominating. Bandages are too frequent and complicated; he brings too many bottles; he is prodigal with the amount of urine, or expectoration, or vomit which he submits for your examination. His cries are too loud; he limps too painfully and laboriously; and a crutch, which often is a valued ally to him, is then used not so much as a support but as an advertisement, the affected limb bearing quite its share of support. I know, or knew, two such men, walking about London with crutches which were veritable croupiers' rakes. Lastly, a clinical thermometer carefully laid by yourself is of extreme value from a diagnostic point of view.

